# Yeast Models for the Study of Amyloid-Associated Disorders and Development of Future Therapy

**DOI:** 10.3389/fmolb.2019.00015

**Published:** 2019-03-22

**Authors:** Sigal Rencus-Lazar, Yasmin DeRowe, Hanaa Adsi, Ehud Gazit, Dana Laor

**Affiliations:** ^1^Department of Molecular Microbiology and Biotechnology, George S. Wise Faculty of Life Sciences, Tel Aviv University, Tel Aviv, Israel; ^2^BLAVATNIK CENTER for Drug Discovery, Tel Aviv University, Tel Aviv, Israel; ^3^Department of Materials Science and Engineering, Iby and Aladar Fleischman Faculty of Engineering, Tel Aviv University, Tel Aviv, Israel

**Keywords:** yeast models, amyloid fibrils, metabolite amyloids, proteinopathy, neurodegenerative diseases, metaboliteopathy, inborn error of metabolism disorders, adenine

## Abstract

First described almost two decades ago, the pioneering yeast models of neurodegenerative disorders, including Alzheimer's, Parkinson's, and Huntington's diseases, have become well-established research tools, providing both basic mechanistic insights as well as a platform for the development of therapeutic agents. These maladies are associated with the formation of aggregative amyloid protein structures showing common characteristics, such as the assembly of soluble oligomeric species, binding of indicative dyes, and apoptotic cytotoxicity. The canonical yeast models have recently been expanded by the establishment of a model for type II diabetes, a non-neurological amyloid-associated disease. While these model systems require the exogenous expression of mammalian proteins in yeast, an additional amyloid-associated disease model, comprising solely mutations of endogenous yeast genes, has been recently described. Mutated in the adenine salvage pathway, this yeast model exhibits adenine accumulation, thereby recapitulating adenine inborn error of metabolism disorders. Moreover, in line with the recent extension of the amyloid hypothesis to include metabolite amyloids, in addition to protein-associated ones, the intracellular assembly of adenine amyloid-like structures has been demonstrated using this yeast model. In this review, we describe currently available yeast models of diverse amyloid-associated disorders, as well as their impact on our understanding of disease mechanisms and contribution to future potential drug development.

## Introduction

A wide range of symptomatically-unrelated diseases are associated with the formation of protein and polypeptide amyloids. These ordered cross-β sheet aggregates have been implicated in the etiology of various disorders, including neurodegenerative diseases, type 2 diabetes (T2D), and systemic amyloidosis (Greenwald and Riek, 2010; Dobson, 2017). Interestingly, the formation of amyloid-like structures by several small metabolites, including nucleobases and amino acids, has also been demonstrated (Adler-Abramovich et al., 2012; Shaham-Niv et al., 2015). The accumulation of these metabolites in various inborn error of metabolism disorders raised the hypothesis that, similar to protein amyloids, metabolite amyloids also play a role in the development of metabolic disorders (Gazit, 2016).

**Figure 1:**
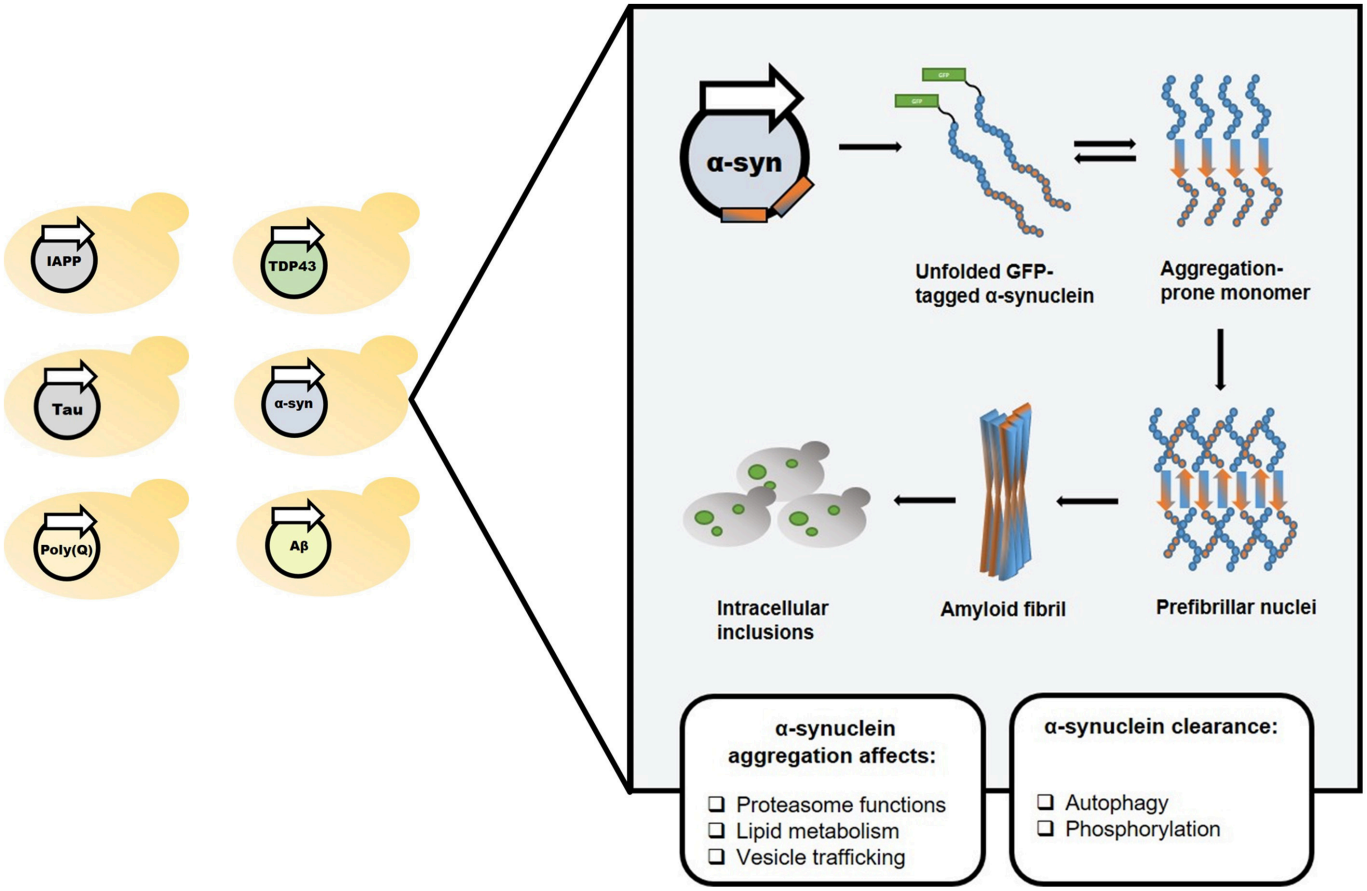
Yeast as a reliable *in vivo* model for studying amyloid-associated disorders. The classical yeast models for amyloid diseases rely on exogenous expression of a protein or polypeptide of interest (left). In the illustrated example, one such model, which utilizes the expression of a GFP-tagged α-synuclein, the amyloidogenic protein underlying Parkinson's disease, has been used to study and characterize the disease's molecular mechanisms.

**Figure 2:**
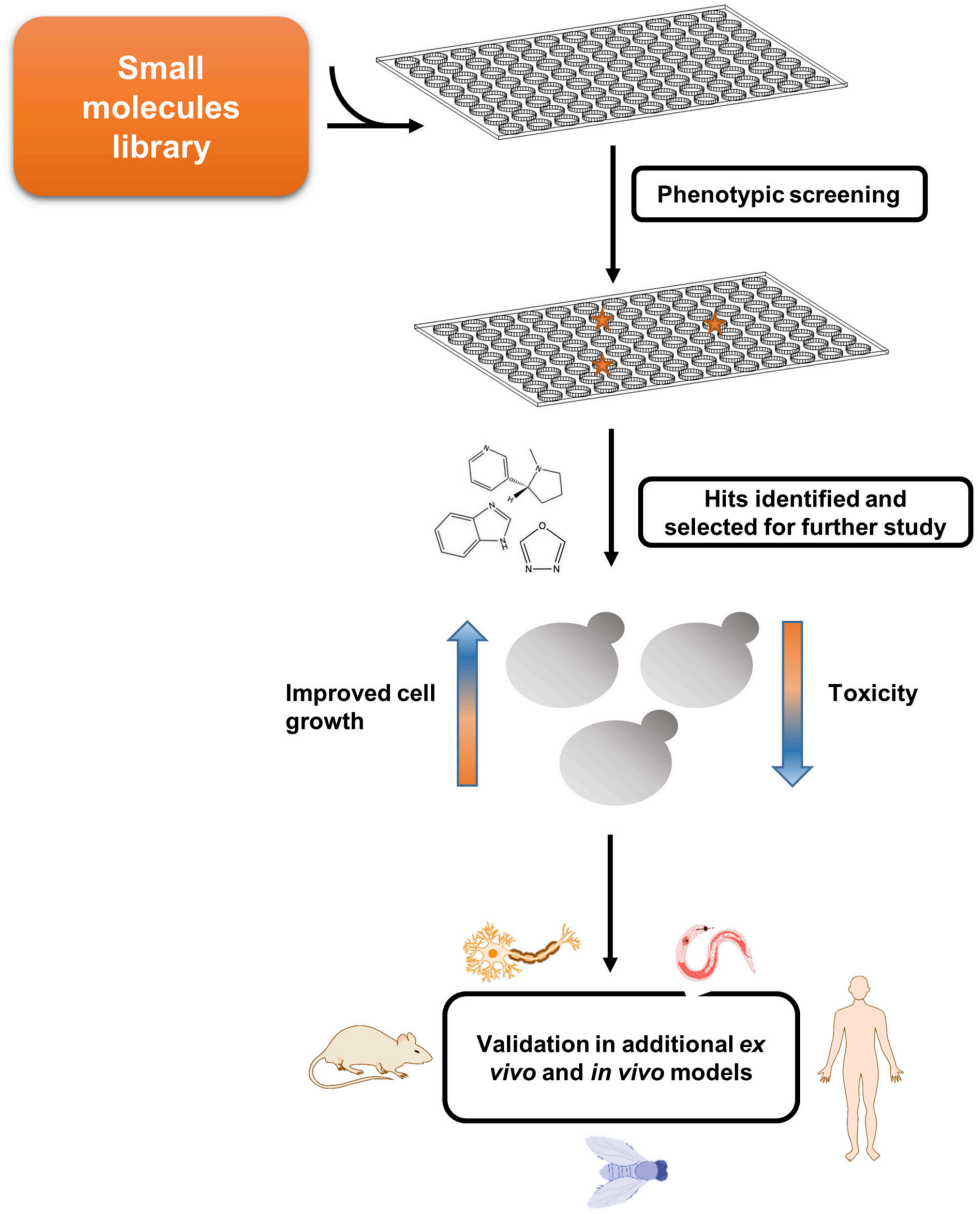
Screen for potential disease-modifying agents employing amyloid disease yeast models. Yeast models can be used for conducting phenotypic screening and relevant hits can be further validated in multicellular organisms.

However, in spite of extensive efforts, no disease-modifying treatment is currently available for amyloid-associated disorders. Aiming to expand our understanding of the underlying molecular mechanisms, and to accelerate the development of therapeutic agents, various model systems have been used. Specifically, in the year 2000, the first yeast model of an amyloid neurodegenerative disease was established (Krobitsch and Lindquist, 2000). Owing to their easy genetic manipulation, highly conserved cellular mechanisms, short life cycle, simplicity, and low-cost, yeast have since become valuable tools for studying amyloid-associated disorders. Furthermore, the discovery of naturally occurring prions in yeast has advanced the study of prion biogenesis and the characterization of common mechanisms for amyloid-associated disorders formation and propagation (Wickner, 1994; Tuite and Cox, 2003). Over the years, the powerful yeast system has facilitated high-throughput genetic screens, allowing to identify various disease-related cellular mechanisms. Yeast also provide a highly useful platform for drug screens aimed at identifying potential therapeutic molecules. Importantly, many of these findings, initially obtained in yeast, were later verified in mammalian systems, thus validating yeast as a reliable model (Figures 1, 2 and Table 1).

**Table 1:** Summary of the main *S. cerevisiae* models for amyloid-associated diseases.

**Disease**	**Main aggregative moiety**	**Mode of gene expression**	**Representative candidate drugs**	**Candidate drugs validation in multicellular organism**	**Representative toxicity modifiers from genetic screens (mammalian homologs)**	**References**
Huntington's Disease	Poly Q	Exogenous	EGCG Actinomycin D C2-8	Human cultured cells Rodents Drosophila	Bna4 (kynurenine 3-mononygenase)	Krobitsch and Lindquist, 2000; Giorgini et al., 2005; Zhang et al., 2005; Ehrnhoefer et al., 2006; Walter et al., 2014; Hofer et al., 2018
Amyotrophic Lateral Sclerosis	SOD1 FUS TDP-43	Exogenous	8-Hydroxyquinolines	_	Pbp1 (Ataxin-2)	Armakola et al., 2011; Ju et al., 2011; Tardiff et al., 2012; Martins and English, 2014; Di Gregorio and Duennwald, 2018
Alzheimer's Disease	Aβ42 Tau	Exogenous	Latrepirdine Clioquinol Dihydropyrimidine-Thiones	Human cultured cells Rodents Nematodes	Yap1801/2 (PICALM)	Vandebroek et al., 2005; Doody et al., 2008; Treusch et al., 2011; Barr et al., 2012; Steele et al., 2013; Amen and Kaganovich, 2016; Park et al., 2016; Seynnaeve et al., 2018
Parkinson's Disease	α-Synuclein	Exogenous	1,2,4-oxadiazoles N-aryl Benzimidazole	Human cultured cells	Rab1 (Ypt1)	Outeiro and Lindquist, 2003; Bhullar et al., 2006; Chung et al., 2013; Tardiff et al., 2013; Vincent et al., 2018
Type 2 Diabetes	IAPP	Exogenous	_	_	Ste24 (ZMPSTE24)	Kayatekin et al., 2018
IEM disorders related to adenine accumulation	Adenine	Endogenous	_	_	_	Laor et al., 2019

Several yeast models of amyloid-associated disorders have recently been comprehensively reviewed (Di Gregorio and Duennwald, 2018; Hofer et al., 2018; Lindström and Liu, 2018). Here, we overview the principal *Saccharomyces cerevisiae* models, focusing on the latest advancements they allowed to achieve. We also present a summary of the promising newly established T2D and adenine accumulation models.

## Huntington's Disease (HD)

The poly-glutamine (poly-Q) disorders comprise a family of genetic neurodegenerative diseases resulting from inherited long stretches of the CAG trinucleotide codon. Specifically, HD is an autosomal dominant neurodegenerative disease caused by multiple CAG repeats in exon 1 of the *huntingtin* gene encoding the N-terminal region of the 350 kD protein huntingtin. In spite of great advancements made since the initial discovery of the gene, no disease-modifying treatment has so far been developed (Caron et al., 2018; Testa and Jankovic, 2019).

Since the establishment of the first HD model in yeast (Krobitsch and Lindquist, 2000), several additional yeast HD models have been described, employing the exogenous expression of poly-Q repeats at variable lengths under the control of different promoters (Hofer et al., 2018). In many cases, expression of the longer glutamine expansions, above a threshold not precisely defined in yeast, resulted in cytotoxicity (Solans et al., 2006; Kaiser et al., 2013; Papsdorf et al., 2015; Ruetenik et al., 2016). Aggregation of expanded poly-Q variants in yeast has been shown to result in impaired endocytosis (Meriin et al., 2003), similar to previous findings in mouse neurons (Kegel et al., 2000). Genetic screens using the yeast HD models have demonstrated the involvement of the kynurenine pathway in poly-Q toxicity in yeast (Giorgini et al., 2005), similar to its well-documented role in mammals (Beal et al., 1990), thus further confirming yeast as a valid HD model. The various aggregates accumulated following expression of poly-Q repeats at different lengths have been comprehensively analyzed using analytical ultracentrifugation with fluorescent detection (Xi et al., 2016). This study demonstrated the formation of multiple types of aggregates, highly diverse in size and structure. This diversity indicates a complex set of molecular mechanisms underlying cell toxicity, currently hindering their full elucidation (Xi et al., 2016; Hofer et al., 2018). Interestingly, these analyses identified mid-sized aggregates to be associated with cell toxicity in yeast (Xi et al., 2016), in accordance with concurrent findings in mouse neuroblastoma cells (Kim et al., 2016), though in mammalian systems this issue is still under debate (Drombosky et al., 2018).

However, several differences between the yeast and mammalian HD models do exist. Notably, structural analysis of a poly-Q expanded N-terminal exon of huntingtin expressed in yeast has recently demonstrated its assembly into unstructured inclusions and, more infrequently, into ordered fibrils. Moreover, no interaction of these aggregates with cellular membranes was detected (Gruber et al., 2018). In contrast, when expressed in mouse or human cultured cells, the same protein assembled into amyloid-like fibrils. These ordered structures interacted with cellular endomembranes, especially the ER membrane, resulting in its deformation (Bäuerlein et al., 2017). Thus, the different structural features of the poly-Q aggregates formed in yeast, as compared to mammalian systems, should be taken into consideration when using these models.

In spite of these differences, the yeast HD models have facilitated major advancements in our understanding of the molecular mechanisms underlying HD etiology, and have served as drug screening platforms. Thus, the yeast models have been employed for screening potential HD-ameliorating drugs, either synthetic (Zhang et al., 2005) or natural (Ehrnhoefer et al., 2006; Walter et al., 2014). These screens allowed to identify compounds, such as (-)-epigallocatechin-3-gallate (EGCG) (Ehrnhoefer et al., 2006) and Actinomycin D (Walter et al., 2014), as novel inhibitors of poly-Q aggregation and toxicity, both further validated in mammalian systems, with the former examined under phase 2 clinical trials. The yeast models have been used to uncover and explore various molecular mechanisms involved in HD etiology, including mitochondrial dysfunction (Solans et al., 2006; Papsdorf et al., 2015; Ruetenik et al., 2016), autophagy (Lu et al., 2014), endocytosis (Meriin et al., 2003), the ubiquitin pathway (Peters et al., 2018), and sumoylation (Ohkuni et al., 2018). Specifically, an yeast HD model has been employed to unravel the role of endoplasmic reticulum (ER)-associated degradation (ERAD) and ER-stress in poly-Q induced cytotoxicity, and the results were further validated in cultured rat neuron-like cells (Duennwald and Lindquist, 2008). These findings provided the basis for unraveling the roles of several factors involved in ER-stress (Carnemolla et al., 2009; Leitman et al., 2014), as well as of the unfolded protein response (Vidal et al., 2012), in HD using mammalian model systems. Recently, LOC14, a small inhibitor of PDI, an ER chaperone elevated during ER-stress, was shown to confer neuroprotective effects and ameliorate ER-stress in a mouse HD model (Zhou et al., 2018). These promising studies exemplify the important contribution of the yeast models to our understanding of the basic molecular mechanisms underlying HD etiology, and the utilization of these findings for identifying potential therapeutic drugs.

## Amyotrophic Lateral Sclerosis (ALS)

ALS, with an estimated prevalence rate of five cases per 100,000 individuals, is a fatal progressive neurodegenerative disease that attacks the motor neurons in the brain and spinal cord. The disease pathology it still an enigma and despite continuous efforts made by the scientific and medical communities, no cure is currently offered to ALS patients. While most cases of ALS are sporadic, about 10% are inherited (familial ALS). Even though no environmental risk factors have so far been identified, several genetic mutations were shown to be implicated mainly in familial ALS, but also in its sporadic form (Oskarsson et al., 2018).

More than ten yeast models expressing different ALS-associated proteins have been established (Damme et al., 2017; Di Gregorio and Duennwald, 2018). The established yeast models include exogenically-expressed mutations in genes involved in RNA metabolism and protein misfolding, such as TAR DNA binding Protein 43 (TDP-43) and fused in sarcoma (FUS), as well as superoxide dismutase 1 (SOD1) (Bastow et al., 2011). TDP-43 and FUS are nuclear proteins that convert, under pathological conditions, into a cytoplasmic aggregated form. One interesting route focuses on studying the involvement of prion-like domains in ALS-linked proteins, including FUS and TDP-43, in the pathogenic mechanisms of the disease in yeast models (Monahan et al., 2018).

A yeast TDP-43 proteinopathy model was successfully generated, conferring cell toxicity, and aberrant accumulation of TDP-43 (Johnson et al., 2008; Armakola et al., 2011; Tardiff et al., 2012). Subsequently, the yeast ortholog of the poly-Q disease gene ataxin 2 (ATXN2), Pbp1, was found to enhance TDP-43 toxicity. This discovery was later confirmed in *Drosophila melanogaster* and in human cultured cells. The human ATXN2 protein was found to be associated in a protein complex with TDP-43 and both are mislocalized in spinal cord neurons of ALS patients. In addition, an intermediate-length poly-Q expansion in the ATXN2 gene was found in ALS patients, suggesting a potential genetic contributor to ALS and a promising target for therapeutic intervention (Elden et al., 2010). FUS is a multifunctional DNA/RNA-binding protein that is predominantly localized in the cell nucleus. A yeast model of human FUS expression was successfully established, showing both cytosolic aggregation and cytotoxicity (Ju et al., 2011), which could be suppressed by several genes, including the heat-shock proteins Hsp40 and Hsp104, several stress granule components, and others. Several of these suppressors have human homologs, indicating their value for studying FUS cytotoxicity in humans (Ju et al., 2011; Sun et al., 2011; Jackrel et al., 2013; Park et al., 2018). SOD1, the first gene identified to be associated with ALS, is a conserved cytosolic reactive oxygen species (ROS) scavenger and the human SOD1 can fully complement the biological *function of yeast SOD1* (Martins and English, 2014). Using this complementation between the two organisms, it was shown that the observed toxicity is correlated with a toxic gain-of-function mechanism involving the loss of SOD1 protein stability. In turn, SOD1 instability can lead to metabolic dysfunction, including loss of vacuolar function and inability to regulate amino acid biosynthesis, which was later validated in a *Caenorhabditis elegans* model of ALS, thus potentially opening a new direction for studying the disease (Bastow et al., 2016).

These models demonstrate the great potential of yeast as a tool to find relevant genes and understand the biological pathways that are involved in ALS pathology, as well as to identify potential therapeutic leads (Figley and Gitler, 2013; Shrestha and Megeney, 2015; Di Gregorio and Duennwald, 2018).

## Alzheimer's Disease (AD)

AD is a progressive disease and the most common form of dementia. In the last decades, the prevalence of AD, especially among the elderly population, has significantly increased. Despite bearing extensive health, social and financial implications, all attempts to counteract this disease have so far failed. The pathophysiology of AD is characterized mainly by the accumulation of intraneuronal tau-containing neurofibrillary tangles in the brain, as well as by the formation of plaques by the Amyloid-β (Aβ) peptide and specifically Aβ42, the pathological isoform of the peptide (Niedowicz et al., 2011).

Yeast models have been utilized for studying these aspects by exogenous expression of the human tau and Aβ proteins. Expressing different clinically-relevant mutant forms of tau in yeast results in many features similar to those observed in patient neurons. These include hyperphosphorylation and other post-translational modifications, conformational changes, partial accumulation into aggregates and recognition by an antibody specific for the tau pathological filaments (Vandebroek et al., 2005; Vos et al., 2011; Heinisch and Brandt, 2016). Yeast models were also extensively used for exploring APP (amyloid precursor protein) processing and secretase activities, as well as Aβ oligomerization and toxicity by expressing either pre-Aβ components (comprising APP substrate and the relevant secretases) or the Aβ peptide (Edbauer et al., 2003; von der Haar et al., 2007; Treusch et al., 2011). Studies in yeast have shed light on the involvement of several pathways, such as the heat-shock response and autophagy, in the pathological outcome of Aβ expression (Caine et al., 2007; Barr et al., 2012). A pioneering work performed by Susan Lindquist and coworkers involved the expression of Aβ42 fused to an ER retention signal, thus allowing to direct the peptide to the secretory pathway, leading to a decrease in cell growth (Treusch et al., 2011). In an attempt to prevent Aβ42 toxicity, a genome-wide screen for modifiers was performed in yeast, leading to the identification of several modifiers, some of which were related to clathrin-mediated endocytosis and were also validated in *C. elegans* and rat hippocampal neurons (Seynnaeve et al., 2018). A follow-up study subsequently demonstrated the inhibition of Aβ42 oligomer formation by one of these modifiers, Yap1802 (Park et al., 2016).

Transnational research has also been applied in an attempt to find potential pharmacological agents for AD therapeutics. Latrepirdine, for example, was shown to enhance autophagy and reduce Aβ42 levels in yeast, mice and human patients, with the former also allowing to understand its mechanism of action (Doody et al., 2008; Barr et al., 2012; Steele et al., 2013). Other studies using drug screens, including FDA-approved drugs, have identified other compounds, some of which have already been validated in mammalians, thereby offering potential novel therapeutic treatments for AD patients (Barr et al., 2012; Matlack et al., 2014; Amen and Kaganovich, 2016; Tardiff et al., 2017).

## Parkinson's Disease (PD)

Affecting over ten million people, PD is the second most common neurodegenerative disease. In spite of extensive efforts, no cure is currently available, raising the need for novel approaches to impede the globally increasing incidence of PD. The accumulation of α-synuclein, a membrane-interacting protein broadly expressed in the brain, followed by the formation of amyloid structures, is a hallmark of PD, as well as of other synucleinopathies (Zhang et al., 2018).

Several PD models have been established in yeast by exogenous expression of various proteins associated with the disease (Menezes et al., 2015). Specifically, while a single copy of a wild-type or mutated GFP-tagged α-synuclein construct did not affect cell growth, two copies of the wild-type or the clinically-relevant A53T mutant-form caused growth inhibition (Outeiro and Lindquist, 2003). Several abnormal phenotypes resulting from α-synuclein expression have been demonstrated in this model, including proteasome impairment, abrogated lipid metabolism, and disrupted vesicle trafficking (Outeiro and Lindquist, 2003). This model was subsequently employed to uncover the involvement of various mechanisms, such as autophagy (Petroi et al., 2012) and phosphorylation (Tenreiro et al., 2014), in α-synuclein clearance, as well as to suggest a mechanism (Kardani et al., 2017) for the previously identified amelioration of PD by nicotine (Quik et al., 2012; Ma et al., 2017). Moreover, the PD yeast model was used to identify N-aryl benzimidazole (NAB), a small drug that ameliorated α-synuclein toxicity by rescuing some of these abnormal phenotypes (Tardiff et al., 2013). NAB was further demonstrated to reverse the pathological phenotypes caused by α-synuclein expression in patient-derived neurons (Chung et al., 2013), thus validating the yeast model as a powerful tool for the identification of potentially therapeutic molecules (Figures 1, 2).

Recently, the PD yeast model was used to identify small molecules ameliorating α-synuclein toxicity, as well as to unravel their cellular target (Vincent et al., 2018). Taking advantage of the growth inhibition phenotype resulting from the induction of α-synuclein expression in yeast, small compounds were screened for growth restoration. A family of molecules containing a 1,2,4-oxadiazole core motif was found to specifically rescue α-synuclein-induced toxicity, while showing no effect on growth inhibition caused by either Aβ or TDP-43. High concentrations of 1,2,4-oxadiazoles also caused growth inhibition of wild-type yeast in correlation to the potency of α-synuclein toxicity rescue, indicating a common underlying mechanism for both activities (Vincent et al., 2018). Two approaches were subsequently applied to identify the cellular target of 1,2,4-oxadiazoles. First, a collection of yeast carrying different point mutations were plated in the presence of YTX-465, the most potent 1,2,4-oxadiazole, at growth-inhibitory concentrations. Resistant colonies were further tested using other drugs, to ensure the identification of specific mechanisms, and fully sequenced to find the mutations. In parallel, a collection of non-essential haploid deletion mutants was plated in the presence of increasing concentrations of YTX-465, allowing to identify mutations conferring either resistance or sensitivity to the compound. Both methodologies indicated the *OLE1* gene, which encodes the sole yeast fatty acid desaturase, as the target of 1,2,4-oxadiazoles. Consistently, YTX-465 treatment reduced fatty acid desaturation in wild-type yeast in a concentration-dependent manner, and supplementation of Ole1 products in the growth medium reduced the YTX-465-mediated growth inhibition. The *OLE1* gene was further demonstrated to be essential for the rescue of α-synuclein toxicity, which did not take place in an *OLE1* deletion mutant. Interestingly, following α-synuclein induction, YTX-465 was found to reduce the levels of triacylglycerols, a major component of intracellular lipid droplets, which were previously shown to accumulate in the yeast PD model (Outeiro and Lindquist, 2003). Moreover, YTX-465 was shown to rescue the impaired intracellular vesicle trafficking caused by α-synuclein expression. Finally, CAY10566, another 1,2,4-oxadiazole compound, was shown to reduce the levels of cell death resulting from the expression of mutated α-synuclein in human cultured neurons, thus validating this family of compounds as potential therapeutic agents (Vincent et al., 2018). This novel study meticulously exemplifies the immense potential of using yeast models of amyloid-related diseases for the identification of candidate drugs and for elucidating the molecular mechanisms underlying their mode of action (Figures 1, 2).

## Type 2 Diabetes (T2D)

T2D is a multifactorial metabolic disease which has turned into a global epidemic. According to the World Health Organization (WHO), T2D comprises the majority of diabetes patients, affecting hundreds of millions worldwide (WHO, 2017). Since in many cases, initial T2D symptoms are mild, diagnosis is often late, emphasizing the crucial need for identifying risk factors and elucidating the molecular mechanisms underlying disease development. Similar to neurodegenerative disorders, such as AD and PD, T2D is also associated with the formation of cytotoxic amyloid fibrils composed of the human islet amyloid polypeptide (IAPP), a 37-residue hormone secreted from the pancreatic islets together with insulin (Westermark, 2005).

A *S. cerevisiae* model for T2D has recently been established (Kayatekin et al., 2018). The model was comprised of yeast overexpressing six IAPP monomers genetically encoded to be linked into a single polypeptide, thereby bypassing the rate limiting nucleation step necessary to initiate amyloid formation (Dobson, 2017). In addition to extremely attenuated growth, the 6xIAPP overexpressing yeast displayed activation of the unfolded protein response and ER stress, similar to other model systems. Moreover, overexpression and deletion screens identified genes involved in proteasome and autophagy regulation as modifiers of IAPP toxicity, consistent with previous models (Costes et al., 2011; Rivera et al., 2014; Kayatekin et al., 2018). The overexpression screen also identified Ste24 as a strong suppressor of IAPP toxicity. Both Ste24 and its human homolog, ZMPSTE24, are peptidases playing a key role in the clearance of trapped polypeptides from the ER translocon (Ast et al., 2016), thus suggesting its clogging by accumulated IAPP oligomers as the underlying mechanism of the ER stress phenotype. Indeed, Ste24 overexpression also relieved the IAPP-induced ER stress. Interestingly, Ste24 overexpression did not suppress the cytotoxicity conferred by either TDP-43 or α-synuclein, thus implicating it in an IAPP-specific mechanism, rather than in a general amyloid phenomenon (Kayatekin et al., 2018).

Based on the high conservation of the yeast Ste24 and the human ZMPSTE24 homologs, the yeast model was utilized to demonstrate their functional conservation in ameliorating IAPP toxicity. Thus, expression of wild-type ZMPSTE24 in yeast cells carrying a chromosomal deletion of Ste24 rescued the toxicity conferred by IAPP overexpression (Kayatekin et al., 2018). The yeast model was further employed to analyze the rescue of IAPP toxicity in the absence of Ste24 by various ZMPSTE24 missense variants naturally found in the human population. Of the 111 examined mutant variants, 14 caused a slower growth phenotype than the wild-type ZMPSTE24, designating them as loss-of-function forms. Though not statistically relevant, these 14 mutants were found to be 2-fold enriched among T2D patients. This study thus exemplifies the application of the yeast model as a tool to identify key molecular mechanisms involved in human amyloid diseases.

## Adenine Accumulation Yeast Model

Inborn error of metabolism (IEM) disorders result from mutations in genes encoding for various metabolic enzymes. Consequently, the corresponding metabolite substrate accumulates, leading to diverse symptoms (Ferreira et al., 2018). However, the molecular mechanisms underlying the development of the specific symptoms of each of these disorders in specific organs are still mostly unknown. Recently, a new paradigm for metabolite accumulation and their putative roles in metabolic disorders was established. Several amino acids and nucleobases, including adenine, have been shown to form archetypical nanofibrils, displaying amyloid-like properties, *in vitro* (Adler-Abramovich et al., 2012; Shaham-Niv et al., 2015). These amyloid-like structures were suggested to be associated with the pathology that underlies several genetic metabolic disorders, thus postulating IEM conditions to constitute yet another group of amyloid-associated disorders (Gazit, 2016). However, studies exploring the formation of amyloid-like assemblies by various metabolites were so far performed mostly *in vitro*. Hence, evidence for the role of metabolite assemblies in an *in vivo* model has been lacking.

Given the high degree of conservation of metabolic pathways across all branches of life, simple models can be established to study the devastating disorders observed upon metabolites accumulation. Thus, aiming to understand the biological relevance and consequences of this metabolite self-assembly, we set out to establish the first *in vivo* yeast model for studying adenine accumulation. In humans, inborn mutations in genes involved in the adenine salvage pathway can lead to the development of several metabolic disorders. Among these, mutations in adenine phosphoribosyltransferase (APRT) and adenosine deaminase (ADA) lead to APRT and ADA deficiency, respectively. The pathology of these disorders involves the accumulation of adenine and its derivatives, which can be toxic or interfere with the normal function of different organs, such as the kidneys and the urinary tract, as well as lead to developmental delays and to severe combined immunodeficiency. In contrast to the yeast models described above, which aim to recapitulate a disease process caused by a toxic gain-of-function, IEM disorders are associated with the lack of a specific enzyme, resulting in the accumulation of upstream substrates. The adenine accumulation model was therefore established through genetic manipulations of the native yeast metabolism. This was achieved by disrupting the APRT and ADA budding yeast orthologs, *APT1* and *AAH1*, leading to high intracellular levels of adenine and thereby allowing to best mimic the metabolite accumulation that underlies the pathology of these disorders *in vivo* (Laor et al., 2019).

**Figure 3:**
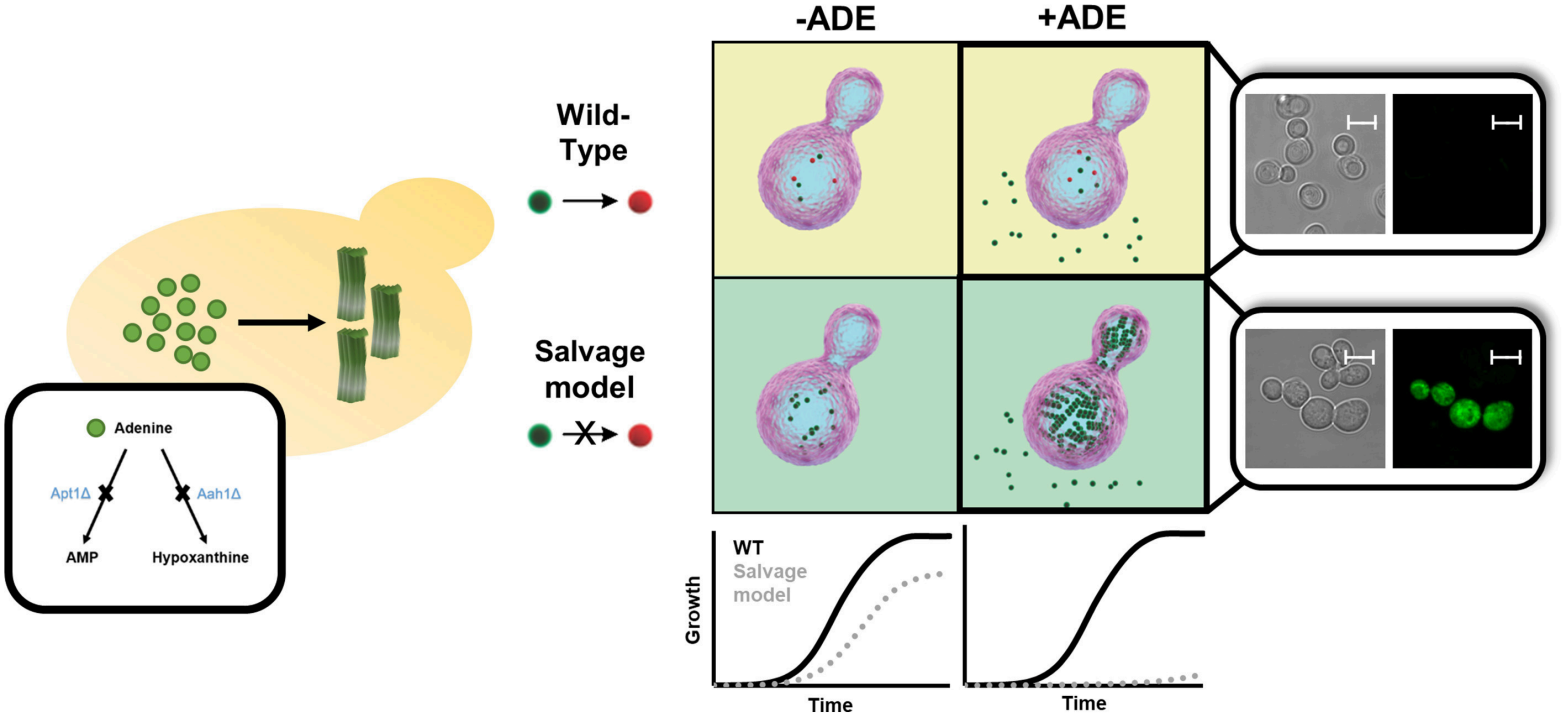
Endogenous *in vivo* model for studying metabolite aggregation. A novel yeast-based model for *in vivo* study of metabolite aggregation phenomena was recently established (Laor et al., 2019). In contrast to the classical yeast models of amyloid-associated diseases, this system does not rely on exogenous expression, but rather on the endogenous disruption of a gene-of-interest, thereby leading to the accumulation, and consequential aggregation of a disease-associated metabolite. Thus, the adenine salvage model, which recapitulates a rare inborn error of metabolism disorder, exhibits similar characteristics to other yeast amyloid models, including a prominent growth phenotype, staining with amyloid-specific dye, etc. Scale bar is 5 μm.

Similar to other yeast models of amyloid-related disorders, the adenine salvage mutant showed a significant reduction in cell growth when the metabolite was present in the medium, along with intracellular accumulation of the metabolite (Figure 3). Above a critical concentration of the nucleobase supplied in the growth medium, a non-linear dose-dependent growth inhibition was detected, consistent with the mechanism of nucleation-growth as observed in the formation of amyloids by the assembly of protein monomers (Dobson, 2017). This toxicity could be rescued by excluding adenine from the medium or by the addition of tannic acid or baicalein, members of the polyphenol family that are known to serve as amyloid inhibitors (Porat and Abramowitz, 2006). Quantification of adenine levels following the addition of tannic acid indicated no change, thus implying that the cell growth inhibition was indeed caused by toxic structures, rather than the mere presence of high levels of adenine. Furthermore, staining with an amyloid-specific dye or an antibody specific for adenine assemblies demonstrated the accumulation of adenine amyloid-like structures inside the yeast cells. To conclude, this study strongly reinforces the reliability of the system as an *in vivo* model to study metabolic disorders that are related to adenine accumulation (Figure 3). This model can serve as a living test tube, allowing to understand the biological relevance and consequences of adenine self-assembly. Altogether, this new model, as previously performed for protein accumulation yeast models (Tardiff et al., 2013; Caron et al., 2018; Vincent et al., 2018), can also serve as a platform for conducting high-throughput screens, potentially assisting in the future discovery of new compounds that may manipulate the self-assembly of adenine amyloid-like structures and can be useful for the treatment of inborn adenine accumulation disorders.

## Conclusions

Yeast models have been efficiently used to study the cellular basis for amyloid-associated degenerative processes. Since their first establishment almost 20 years ago, yeast models of amyloid-related diseases have proven a highly instrumental system, allowing to elucidate key molecular mechanisms taking part in these ailments. The facile genetic manipulation and the diverse genetic and biochemical tools available in yeast have facilitated high-throughput screens, allowing to identify central factors effecting amyloid formation and cytotoxicity. Many of these mechanisms were later shown to be conserved in mammalian systems. Moreover, drug screens, which are easily and rapidly achievable in yeast, have allowed to identify several promising drug candidates. Thus, the yeast PD model has been utilized to identify 1,2,4-oxadiazoles as potential drug candidates (Vincent et al., 2018), and the involvement of ER-stress in HD, originally demonstrated in yeast (Duennwald and Lindquist, 2008), has been employed to identify LOC14 as a potential therapeutic molecule in a mouse model (Zhou et al., 2018). Hence, in spite of the inherent limitations of a unicellular system, the yeast amyloid models have provided valuable insights, which might have been unattainable using other model organisms. The newly established T2D and adenine accumulation models (Kayatekin et al., 2018; Laor et al., 2019) are expected to similarly provide mechanistic insights into the corresponding diseases, as well as facilitate the identification of potential drugs.

While *proteinopathy* yeast models exist for almost two decades, the first “*metaboliteopathy*” yeast model has recently been established, thus providing exciting new possibilities for the research of metabolic disorders (Figure 3) (Laor et al., 2019). Future studies employing yeast and other *in vivo* models can provide great benefit to our understanding of the main factors that take part in *proteostasis* processes, as well as in *metabostasis* mechanisms. As already widely implemented in some of the systems described above, studies in yeast models should be further validated in multicellular organisms to serve as basis for a potential cure (Figures 1, 2 and Table 1).

Interestingly, an important biological function of amyloid-like protein aggregates formation and clearance during meiosis has been recently demonstrated in yeast, thus exemplifying their utility for studying natural mechanisms of amyloid processing (Carpenter et al., 2018). Moreover, the formation of reversible functional amyloids by the yeast pyruvate kinase Cdc19 under stress conditions has been recently reported (Cereghetti et al., 2018). This line of studies raises the intriguing possibility of protein aggregation as a general physiological, rather than pathological, mechanism. Further studies should reveal whether physiological and pathological aggregates share common formation and clearance mechanisms, thus potentially allowing to harness natural clearance mechanisms for the treatment of amyloidogenic conditions (Carpenter et al., 2018).

Similar to its key contribution to our understanding of fundamental phenomena, such as cell cycle (Hartwell et al., 1973), telomeres (Janis et al., 1984; Zakian, 2009), and autophagy (Zimmermann et al., 2016), we expect the employment of yeast models to lead to a similar progress in deciphering the molecular mechanisms underlying amyloid-associated diseases, as well as to the identification of suitable drug molecules (Zimmermann et al., 2018).

## Author Contributions

SR-L, EG, and DL wrote the manuscript. YD and HA were involved in the generation of the table and figures and critically revised the manuscript. All authors contributed to the conception of this review article.

### Conflict of Interest Statement

The authors declare that the research was conducted in the absence of any commercial or financial relationships that could be construed as a potential conflict of interest.
